# Characterization of translocon proteins in the type III secretion system of *Lawsonia intracellularis*

**DOI:** 10.1186/s13567-023-01243-0

**Published:** 2023-11-22

**Authors:** Beibei Huang, Zihe Zhu, Yimin Dai, Chengxian Yan, Jingyu Xu, Lingling Sun, Qinghua Zhang, Xuejiao An, Fenju Lai

**Affiliations:** 1 School of Bioscience and Bioengineering, Nanchang Key Laboratory of Fermentation Application Technology, Jiangxi Agricultural University, Nanchang, 330045 China; 2 School of Animal Science and Technology, Jiangxi Agricultural University, Nanchang, 330045 China; 3 Jiangxi Engineering Laboratory for the Development and Utilization of Agricultural Microbial Resources, Nanchang, Jiangxi China

**Keywords:** Proliferative enteropathy, *Lawsonia intracellularis*, type III secretion system, translocon proteins

## Abstract

*Lawsonia intracellularis*, the etiologic agent of proliferative enteropathy (PE), is an obligate intracellular Gram-negative bacterium possessing a type III secretion system (T3SS), which enables the pathogen to translocate effector proteins into targeted host cells to modulate their functions. T3SS is a syringe-like apparatus consisting of a base, an extracellular needle, a tip, and a translocon. The translocon proteins assembled by two hydrophobic membrane proteins can form pores in the host-cell membrane, and therefore play an essential role in the function of T3SS. To date, little is known about the T3SS and translocon proteins of *L. intracellularis*. In this study, we first analyzed the conservation of the T3S apparatus between *L. intracellularis* and *Yersinia*, and characterized the putative T3S hydrophobic major translocon protein LI1158 and minor translocon protein LI1159 in the *L. intracellularis* genome. Then, by using *Yersinia pseudotuberculosis* as a surrogate system, we found that the full-length LI1158 and LI1159 proteins, but not the putative class II chaperone LI1157, were secreted in a − Ca^2+^ and T3SS-dependent manner and the secretion signal was located at the N terminus (aa 1–40). Furthermore, yeast-two hybrid experiments revealed that LI1158 and LI1159 could self-interact, and LI1159 could interact with LI1157. However, unlike CPn0809 and YopB, which are the major hydrophobic translocon proteins of the T3SS of *C. pneumoniae* and *Yersinia*, respectively, full-length LI1158 was non-toxic to both yeast and *Escherichia coli* cells, but full-length LI1159 showed certain toxicity to *E. coli* cells. Taken together, despite some differences from the findings in other bacteria, our results demonstrate that LI1158 and LI1159 may be the translocon proteins of *L. intracellularis* T3SS, and probably play important roles in the translocation of effector proteins at the early pathogen infection stage.

## Introduction

*Lawsonia intracellularis* is an obligate intracellular Gram-negative bacterium isolated in 1993 and the etiologic agent of proliferative enteropathy (PE), an infectious disease macroscopically characterized by thickening of the intestinal epithelium due to enterocyte proliferation [1, 2]. After an initial close association with the cell membrane of enterocytes, *L. intracellularis* is endocytosed into host cells and inhibits their maturation, and then the infected host cells will continue to undergo mitosis and proliferation and finally form hyperplastic crypts [2, 3]. Mild to severe diarrhea are the major clinical signs observed in infected animals [3]. Despite decades of research on this pathogen, current understanding of its molecular pathogenesis is still rather limited due to its obligate intracellular nature, genetic intractability, and lack of an in vitro bacterial model [4, 5].

The type III secretion system (T3SS), a well-characterized system, plays significant roles in the pathogenicity of many Gram-negative bacterial pathogens [6]. Currently, several enteropathogenic pathogenic bacteria, such as *Yersinia*, *Salmonella* spp., *Shigella* spp., and enteropathogenic and enterohemorrhagic *Escherichia coli* (EPEC/EHEC), have been well characterized and are known to utilize T3SS for infection of their hosts to cause a variety of infectious diseases [6]. The Ysc-Yop T3SS from *Yersinia* was the first characterized T3SS and is often regarded as the archetype [7, 8]. The T3S components and its substrates, namely the Yop effector proteins (YopH, YopO/YpkA, YopP/YopJ, YopE, YopM, and YopT), are all encoded by a 70-kb virulence plasmid called pYV in *Y. enterocolitica* [9]. In vitro, the need for contact with eukaryotic cells can be circumvented by chelating Ca^2+^ ions with agents such as EGTA or oxalate [10]. Since the T3SS has a conserved structural framework across different animal bacterial pathogens, *Yersinia* has been successfully employed as a surrogate system to identify novel T3SS substrates for genetically intractable strains [11].

T3SS is a syringe-like apparatus comprising 20–25 proteins, which compose a base, an extracellular needle, a tip complex, and a translocator or translocon (used throughout this article). The tip protein (LcrV of the Ysc family) complex serves as a platform for the assembly of the translocon, which forms a translocon pore in the host cell membrane [6, 7, 12]. The translocon pore is assembled by two hydrophobic membrane proteins, a major translocon protein and a minor translocon protein determined according to their relative sizes compared with each other [12]. Each pair of major and minor translocon proteins share a common class II chaperone, whose coding genes are localized next to the genes encoding the translocons and the tip proteins, and are expressed from one operon [12]. For the Ysc family in *Yersinia*, the needle protein YscF and tip protein LcrV are indispensable for the translocation of effector proteins into host cells [13, 14]. LcrH/SycD, an archetypal class II chaperone, binds to the translocon proteins (YopB and YopD, the major and minor translocon protein) and prevents their premature folding and homo- or hetero-oligomerization in the cytosol of bacterial cells [15].

Most proteins composing the T3S apparatus are conserved among species possessing a T3SS. Genes encoding the T3S apparatus have been identified in the complete genome of *L. intracellularis* strain PHE/MN1-00, and the expression of some components has been verified by RT-PCR and serological response during infection [2, 16]. Moreover, an operon genetically related to the Salmonella pathogenicity island 2 (SPI2) and composed of three genes, including a chaperone (LI1157, PcrH), an effector protein (LI1158, SseC), and a hypothetical protein LI1159 (referred to as SseD), was found to be highly expressed in the cytoplasm of porcine enterocytes infected with *L. intracellularis* [17]. Combined with findings in other similar bacteria, it can be inferred that this T3SS may be crucial for intracellular development and probably plays a significant role in the virulence of this unusual pathogen. However, the experimental identification of T3S components and substrates of *L. intracellularis* is largely hampered by the difficulty in culturing of the bacteria under axenic (cell-free) conditions, and the best method for the identification may be the use of a T3S surrogate system.

In this study, we first bioinformatically and experimentally characterized LI1158 and LI1159 as putative hydrophobic translocon proteins. Furthermore, by using the yeast two-hybrid system, we found that LI1158 and LI1159 can self-interact, and LI1159 can interact with LI1157. These results are consistent with the assumption that LI1158 and LI1159 are the major and minor translocon proteins of *L. intracellularis* T3SS, respectively, and both play certain roles in the target-cell attachment and invasion of *L. intracellularis*. However, we also obtained some different results compared with the findings in other bacteria. Our yeast two-hybrid (Y2H) assay showed that LI1158 had no interaction with LI1159 and LI1157. The expression of LI1158 resulted in no strong inhibitory effect on the growth of yeast cells and *E. coli* cells, but LI1159 expression obviously inhibited the growth of *E. coli* cells. These results improve our understanding of the T3SS translocon proteins in *L. intracellularis* and will facilitate the development of more specific serological diagnostic assays and effective vaccines to control PE outbreak.

## Materials and methods

### Strains, cell lines, and media

*E. coli* DH5α (TransGen Biotech, Beijing, China) was used for plasmid amplification, serial dilution patch tests, and growth curve assay. The strain was propagated in Luria–Bertani (LB) supplemented with 100 μg mL^−1^ ampicillin at 37°C with agitation or on LB agar plates. The *Y. enterocolitica* T3S-proficient MRS40_ΔyopHOPEM_ and T3S-deficient MRS40_ΔyscF_ were used for T3S secretion assays, and MRS40_ΔyopHOPEM_ was also used for serial dilution patch tests and growth curve assay. *Yersinia* was routinely grown in brain heart infusion (BHI; TransGen Biotech) with 200 µg mL^−1^ ampicillin to select the expression vectors at 30°C with agitation or on BHI agar plates.

The *Saccharomyces cerevisiae* strain Y2H gold (*MAT*a, *trp1*-901, *leu*2-3, 112, *ura*3-52, *his*3-200, *gal4*Δ, *gal80*Δ, LYS2::GAL1_UAS_–Gal1_TATA_–*His*3 GAL2_UAS_–Gal2_TATA_–*Ade2*, URA3::MEL1_UAS_–Mel1_TATA_-AUR1-C *MEL1*) (Clontech, Takara Bio USA, Inc., Mountain View, CA, USA) was used for Y2H experiment and W303-1A (*MAT****a**** ade*2-1 *ura*3-1 *his*3-11,15 *trp*1-1*, leu*2-3,112 *can-*100) (Clontech) was use for growth inhibition assay. Both strains were grown at 30°C in yeast extract peptone dextrose (YPD) or synthetic dropout medium (SCM minus).

The human embryonic kidney (HEK) 293 T cell line (Thermo Fisher Scientific, San Jose, CA, USA) was cultured in Dulbecco’s modified Eagle’s medium (DMEM, Invitrogen Tech, Carlsbad, CA, USA) supplemented with 10% (v/v) fetal bovine serum (FBS, Gibco, Waltham, MA, USA), 1 mmol L^−1^ glutamine, and 100 U mL^−1^ penicillin and streptomycin.

### Plasmid construction

The primers and plasmids used in this study are listed in Tables 1 and 2, respectively. The DNA fragments encoding full-length LI1157-LI1159 amplified from genomic DNA extracted from *L. intracellularis*-positive porcine ileal mucosa with a C-terminal hemagglutinin (HA) epitope tag fragment were separately cloned into the *EcoR* I/*Hind III* sites of the pBAD24 vector. The first 40 amino acids (aa) of LI1158 and 1159 were cloned into frame with the C-terminal amino acids of YopE (YopE_Δ15_) in the previously constructed pBAD24-YopE-HA plasmid, respectively [18]. Plasmids were introduced into *Y. enterocolitica* by electroporation.

**Table 1:** **Primers used in this study**.

Primer	Sequence 5′–3′ (restriction enzyme sites are underlined)
pBAD24-LI1157-F	GTTTTTTTGGGCTAGCAGGAGGAATTCATGGTTATGAAACAAGAG
pBAD24-LI1157-R	ATCTGGTACGTCGTATGGGTAGTCGACTTGCTTTACTCCTCCAGA
pBAD24-LI1158-F	GTTTTTTTGGGCTAGCAGGAGGAATTCATGACAAATTTTGGAGAT
pBAD24-LI1158-R	ATCTGGTACGTCGTATGGGTAGTCGACCTCACGTGCACCACGTTG
pBAD24-LI1158_N40_-R	GCTAGATCCTGACACAGAACCACCTTGCTCTTTAGG
pBAD24-LI1159-F	GTTTTTTTGGGCTAGCAGGAGGAATTCATTTTGGAGAAGACTATC
pBAD24-LI1159-R	ATCTGGTACGTCGTATGGGTAGTCGACCGCCAGAATACGCTGTGT
pBAD24-LI1159_N40_-R	CGCTGCTAGATCCTGACACAGATTTAGCAGGTTGTCCACCAGAGGA
pRS416-GAL1-LI1158-F	ATTACAAGGATGACGATGACAATGGCGGAGGAGCGGCCGCGATGACAAATTTTGGAGAT
pRS416-GAL1-LI1158-R	GCGTGAATGTAAGCGTGACATAACTAATTACATGACTCGAGTTACTCACGTGCACCACG
pSIN-LI1158-F	GGATCCCCGGACGAATTCTTCGAAACCATGACAAATTTTGGAGAT
pSIN-LI1158-R	AGCAGCAGCGGTTTCTTTGCTAGC CTCACGTGCACCACGTTG
pGADT7-LI1157-F	CCGGAATTCATGGTTATGAAACAAGAGC
pGADT7-LI1157-R	CCCTCGAGTTGCTTTACTCCTCCAGAGAGAAT
pGADT7-LI1158-F	GCCATGGAGGCCAGTGAATTCATGACAAATTTTGGAGATATAAG
pGADT7-LI1158-R	ACGATTCATCTGCAGCTCGAGTTACTCACGTGCACCACG
pGBKT7-LI1158-F	CATATGGCCATGGAGGCCGAATTCACAAATTTTGGAGATATA
pGBKT7-LI1158-R	TGCGGCCGCTGCAGGTCGACGGATCCCTTACTCACGTGCACCACG
pGADT7-LI1159-F	CCGGAATTCATTTTGGAGAAGACTATCA
pGADT7-LI1159-R	CCCTCGAGCGCCAGAATACGCTGTGTTGTTGC
pGBKT7-LI1159-F	CATATGGCCATGGAGGCCGAATTCATTTTGGAGAAGACTATC
pGBKT7-LI1159-R	CGGCCGCTGCAGGTCGACGGATCCTTACGCCAGAATACGCTG

**Table 2:** **Plasmids construction in this study**.

Plasmids	Genotype/description
pBAD24-LI1157-HA	araC promoter, LI1157-HA, ampicillin
pBAD24-LI1158-HA	araC promoter, LI1158-HA, ampicillin
pBAD24-LI1159-HA	araC promoter, LI1159-HA, ampicillin
pBAD24-LI1158_N40-_YopE_Δ15_-HA	araC promoter, LI1158_N40-_YopE_Δ15_-HA, ampicillin
pBAD24-LI1159_N40-_YopE_Δ15_-HA	araC promoter, LI1159_N40-_YopE_Δ15_-HA, ampicillin
pRS416-GAL1-LI1158	Gal1 promoter, 3 × Flag-LI1158, ampicillin
pSIN-EF1-puro-SFB-LI1158	EF1α promoter, LI1158-S-tag-2 × Flag-SBP, ampicillin
pGADT7-LI1157	T7 promoter, AD-HA-LI1157, ampicillin
pGADT7-LI1158	T7 promoter, AD-HA-LI1158, ampicillin
pGADT7-LI1159	T7 promoter, AD-HA-LI1159, ampicillin
pGBKT7-LI1158	T7 promoter, BD-c-Myc-LI1158, Kanamycin
pGBKT7-LI1159	T7 promoter, BD-c-Myc-LI1159, Kanamycin

The DNA fragment encoding full-length LI1158 was cloned into the galactose-inducible yeast expression vector pRS416-GAL1, Y2H bait expression vector pGADT7 (Clontech), and prey expression vector pGBKT7 (Clontech), respectively. The DNA fragment encoding full-length LI1159 was cloned into the pGADT7 and pGBKT7, respectively. The DNA fragment encoding full-length LI1157 was cloned into the pGADT7. Yeast transformation was performed using the lithium acetate method and yeast whole cell lysates were prepared from yeast cells grown to log phase in selective medium and resolved by SDS­PAGE.

The DNA fragment encoding full-length LI1158 was cloned into the mammalian expression vector psin-SFB (psin-EF1 plasmid, tagged with S Protein-Flag-streptavidin-Binding peptide). Plasmid DNA was harvested from *E. coli* using an endotoxin-free DNA isolation kit (Qiagen, Valencia, CA, USA) and transfected into HEK293T cell monolayers by using Lipofectamine^®^ 2000 reagent (Invitrogen, Carlsbad, CA, USA). At 48 h after transfection, cells were lysed in lysis buffer (phosphate 20 mM, NaCl 0.5 M, imidazole 20 mM, Triton X-100 2%, pH 7.4) at 4 °C for 1 h, and the purified lysates were resolved by SDS–PAGE.

### Bioinformatic analysis

BLAST algorithms were used to identify sequence similarity between proteins. TMHMM 2.0 and InterPro were used to locate Transmembrane domain (TM) and coiled-coil (CC) domain in full-length LI1158/LI1159 proteins.

### *Y. enterocolitica* T3S secretion assays

Secretion assays in *Y. enterocolitica* were performed as described previously [19]. The supernatant and remaining pellet were separated by centrifugation and analyzed by SDS–PAGE. Proteins were transferred to a polyvinylidene difluoride (PVDF) membranes, which were probed with rat monoclonal anti-HA antibody (CST; 1:2000). Immunoblot detection was performed with secondary antibodies directed against rabbit antibodies and conjugated to horseradish peroxidase (Thermo Fisher) and enhanced chemiluminescence (ECL) chemiluminescent substrate (Proteintech, Wuhan, China).

### Yeast growth assays

Yeast growth assays were performed as described previously [20]. Briefly, overnight cultures of recombinant strains grown in SD-Ura medium were washed, and absorbance values were normalized to an optical density of 1.0 at OD_600_. Each strain was serially tenfold diluted for four times and spotted (4 µL) onto repression (2% glucose) or induction (2% galactose) solid selective medium. Yeast cultures were then incubated at 30°C for 2–4 days.

### Preparation of yeast extracts and immunoblot analysis

The yeast extract preparation and immunoblot analysis were performed as described previously [20]. Mouse monoclonal anti-Flag antibodies (Proteintech) were used for immunoblotting. Immunoblot detection was performed with secondary antibodies directed against mouse antibodies and conjugated to horseradish peroxidase (Thermo Fisher) and ECL chemiluminescent substrate (Proteintech).

### Extract preparation and immunoblot analysis in mammalian cells

The mammalian cell extract preparation and immunoblot analysis were performed as described previously [20]. Briefly, asynchronously growing HEK293T cells were seeded at 2.5 × 10^5^ cells/well in a 6-well tissue culture plate and serum-starved overnight. Two micrograms of plasmid DNA was transfected into HEK293T cells with LipofectamineTM 2000 (Invitrogen) according to the manufacturer’s protocol. Cells were washed with ice-cold PBS containing 1 mM Na_3_VO_4_ and 10 mM NaF and were then lysed with 300 µL of RIPA buffer containing protease inhibitors. Equal volumes of samples were subjected to SDS-PAGE. Proteins were transferred to PVDF membranes, which were probed with mouse monoclonal anti-Flag antibodies (Proteintech). Immunoblot detection was performed with secondary antibodies directed against mouse antibodies and conjugated to horseradish peroxidase (Thermo Fisher) and ECL chemiluminescent substrate (Proteintech).

### Yeast-two-hybrid assays

Combinations of plasmids (bait and prey) were transformed into *S. cerevisiae* strain Y2H Gold, and the interaction was tested by serial dilution patch tests on the selective medium (Leu-, Trp-; growth control) and low-stringency medium (Leu-, Trp-, His-). The plasmids pGBKT7-p53 (human protein p53) and pGADT7-T (large T antigen of SV40) as the positive control and pGBKT7-Lam (Lamin C) and pGADT7-T as the negative control were co-transformed into yeast cells.

## Results

### The core T3S apparatus is conserved in *L. intracellularis*

Genes encoding the T3S apparatus have been identified and named in the complete genome of strain PHE/MN1-00 (accession number AM_180252), which are scattered throughout *L. intracellularis* genome (Table 3). In order to better analyze the conserved nature of T3SS, we blasted the sequence of T3S components in *L. intracellularis* PHE/MN1-00 and *Y. enterocolitica* pYVe8081 (accession number NC_008791.1) with the blast analysis tool at the National Center for Biotechnology Information (NCBI). Although approximately 25 proteins are required to build the T3S apparatus, only genes encoding the core of T3S apparatus proteins conserved in different bacteria and partial orthologous proteins with low sequence conservation have been annotated in the complete genome of *L. intracellularis* strain PHE/MN1-00 (Table 3) [7]. BLASTP analysis of these proteins revealed that their sequence similarity to the orthologous proteins in *Y. enterocolitica* T3SS was 24–66%, which was comparable to the conservation among different T3SS-containing pathogens (Table 3). Hence, it can be speculated that the T3SS of *L. intracellularis* may be crucial for its intracellular development and probably plays a significant role in the virulence of this unusual pathogen.

**Table 3:** **Orthologous proteins between** ***L. intracellularis* and** ***Y. enterocolitica* in type 3 secretion system**.

*Yersinia* spp.	*L. intracellularis*	Identities (%)	Structure/function
Injectisome proteins (basal structure) that share sequence similarity			
YscR	LI0537	54	Basal structure, inner-membrane protein
YscQ	LI0538	23	Putative C ring?
	LI1166	37.3	Putative C ring
YscN	LI0540	66.8	ATPase, ring with sixfold symmetry
YscV	LI0548	51.4	Basal structure, inner-membrane protein
YscS	LI0549	64.3	Basal-structure, inner membrane protein
YscT	LI0550	54	Basal structure, inner-membrane protein
YscU	LI0551	39	Basal structure, inner-membrane protein involved in substrate specificity switching
YscC	LI1160	32	Secretin, outer-membrane protein ring subunit
YscJ	LI1163	34	MS ring, lipoprotein
Orthologous proteins with low sequence conservation			
YopB	LI1158	27	Major translocon protein
YopD	LI1159	24	Minor translocon protein
YscL	LI0541	29	Interacts with the ATPase and the putative C ring Could be tethering the ATPase in the export channel
	LI1164	24	
LcrH/SycD	LI1157	29	Chaperon of translocon proteins

### LI1158 and LI1159 possess the characteristics of T3S major and minor translocon proteins

**Figure 1:**
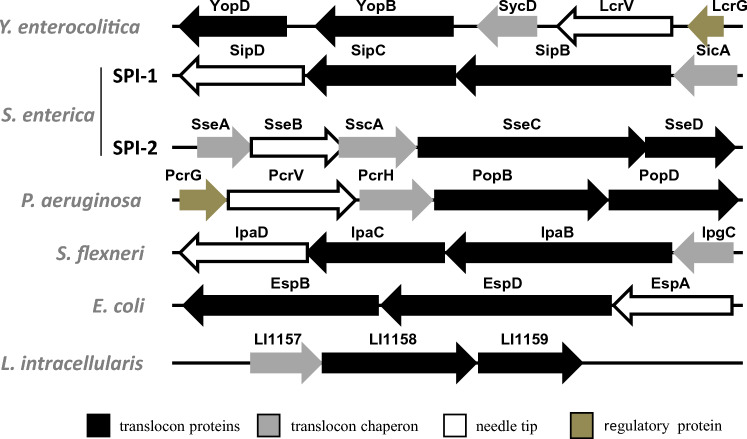
**Putative components of the T3SS encoded by the LI1157–1159 operon in *L. intracellularis* identified by comparison with other bacteria.** The arrangement of T3SS translocon operons is highly conserved in pathogenic Gram-negative bacteria, with several well characterized archetypal translocon operons serving as a basis for comparison. LI1157–LI1159 have the characteristics of the typical T3SS translocon operon structure found in a variety of pathogenic bacteria. The role of the putative proteins is indicated using a color code, with arrows denoting gene transcription orientation.

The assembly of T3S apparatus requires the tip complex and the translocon, which are also T3SS substrates, and the translocon proteins form pores in the plasma membrane of the target host cells [12]. As shown in Figure 1, the translocon operons of different bacterial species generally encode two hydrophobic translocon proteins and a hydrophilic tip protein, which are co-expressed with a class II chaperone and, in some cases, with other regulatory proteins. Similar to the case of these translocon operons, LI1158 (the putative major translocon protein) was co-expressed with LI1157 (the putative chaperone of translocon proteins) and LI1159 (the putative minor translocon protein) from one operon in *L. intracellularis* genome. However, different from other bacteria, both LI1156 and LI1160, the LI1157 and LI1159 neighboring protein, did not encode the tip protein. Instead, LI1156 encoded a hypothetical protein without conservation with LcrV, while LI1160 encoded YscC, an outer-membrane protein ring subunit of T3SS. Given that LcrV is needed for the insertion of a translocon into the target cell membrane, it is surprising that genome analysis did not annotate the LcrV orthologue in *L. intracellularis*, which may be attributed to the low sequence homology of *L. intracellularis* LcrV with its homologous gene. In future studies, it would be interesting to analyze the LcrV protein and its interaction with LI1158 and LI1159 in more detail.

**Figure 2:**
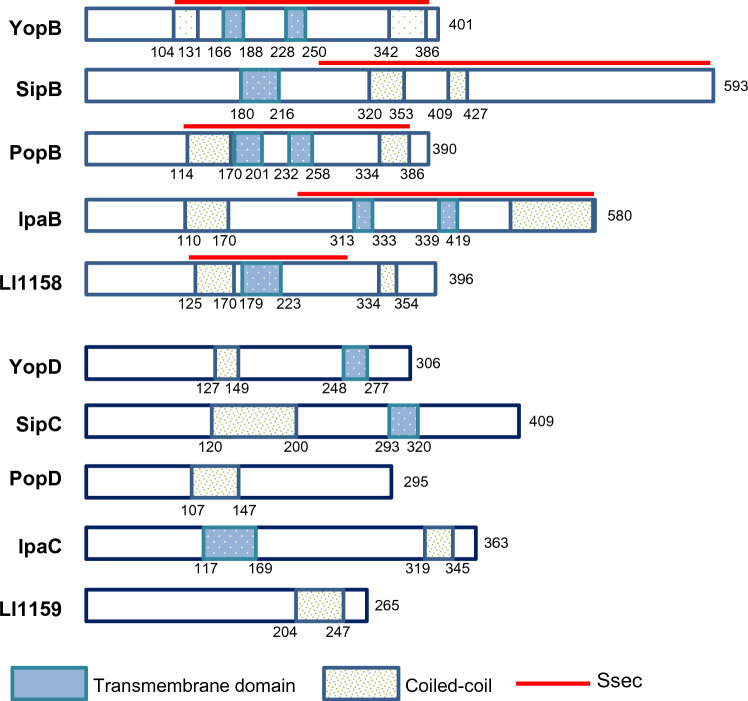
**Domain maps of the minor and major translocon proteins.** Comparison of *L. intracellularis* LI1158/LI1159 with the major/minor translocon proteins from other Gram-negative bacteria. Numbers indicate amino acid positions. The distribution of predicted TM, CC domains and “SseC-like family” within LI1158/LI1159 and the other major/minor translocon proteins is shown schematically. The TM and CC domains of LI1158/LI1159 were predicted by TMHMM 2.0 and Marcoil, respectively. The SseC domain of LI1158/LI1159 was identified by Scansite 4.0 searches. The TM and CC domains of other Gram-negative bacteria were modified from reference [12].

Sequence analysis revealed that LI1158 shared 27% identity with the YopB of *Yersinia* spp*.* (Table 3). Further bioinformatic analysis suggested that LI1158 harbors two TM (aa 179–200, 206–223) in its central region, which are flanked by two CC domains (aa 125–170, 334–354) (Figure 2). In addition, an “SseC (SPI-2 effector of *Salmonella*)-like family” domain was identified in the C-terminal portion of LI1158 (aa 120–308) (30% identity; 52% similarity with a 38% coverage). SseC domains were found in the major hydrophobic translocon proteins of all bacterial T3SSs considered in this study, while they were absent in most minor hydrophobic translocon proteins (Figure 1) [21, 22]. In general, the SseC domains of the translocon proteins displayed some common features, including two or more hydrophobic domains (HD) combined with one or more CC domains. In most cases, the SseC and hydrophobic domains encompassed the C-terminal end of the protein (Figure 2). Furthermore, the PxLxxP motif, which has been identified in translocon proteins and is essential for binding LcrH_1 and a chaperone in *C. pneumoniae,* was also found in LI1158 (aa 105–110) [22, 23]. Thus, LI1158 exhibited structural similarities to the T3SS major hydrophobic translocon protein.

Sequence analysis demonstrated that LI1159 shared 24% identity with the YopD of *Yersinia* spp. (Table 3). Further bioinformatic analysis suggested that LI1159 harbors one CC domain (aa 204–247) at the C terminus (Figure 2). However, different from other minor translocon proteins which were predicted to contain one TM, LI1159 had no TM. Given that the TM is essential for the insertion of minor translocon proteins into the target cell membrane, further research is needed to determine which amino acids are involved in the insertion of LI1159 into the cell membrane.

Taken together, these bioinformatics analysis results suggested that despite the difference between *L. intracellularis* and other Gram-negative bacteria, LI1158 and LI1159 may function as the major and minor translocon proteins of T3SS in *L. intracellularis*.

### LI1158 and LI1159 are secreted in a *Y. enterocolitica* surrogate system with the secretion signal located at the N terminus

**Figure 3:**
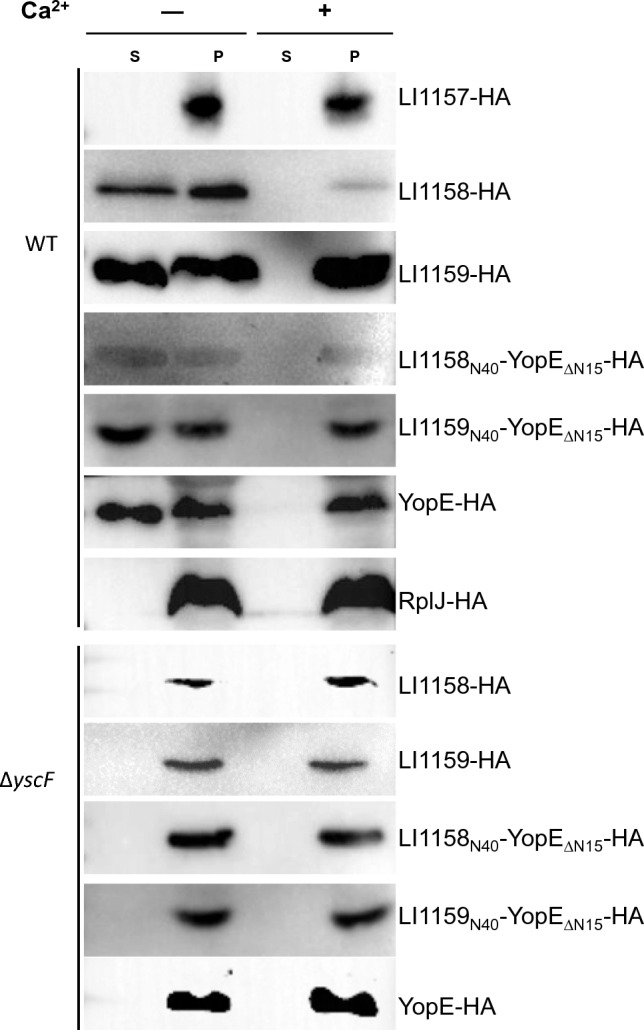
**LI1159 and LI1159 are the substrates of the *Yersinia* type 3 secretion system.** T3SS-competent *Y. enterocolitica* MRS40_∆YopHOPEM_ and T3SS-null *Y. enterocolitica* MRS40_∆yscF_ expressing various HA-tagged proteins (LI1157, LI1158, LI1159, LI1158_N40_-YopE_∆N15_, LI1159_N40_-YopE_∆N15_, RplJ, YopE) were cultivated in BHI media under either T3SS-repressive (+ Ca^2+^) or -inductive (− Ca^2+^) conditions. The bacteria were induced to express the T3SS and transgenes by adding 0.2% L-arabinose and incubated at 37 °C for 6 h. Equal amounts of bacteria cultures were separated into cell-free culture supernatants (S) and whole-cell pellets (P), and samples corresponding to 0.10 OD_620_ per mL for S and 0.02 OD_620_ per mL for P were analyzed on 12% (w/v) polyacrylamide gels. Specific proteins were detected by immunoblot with anti-HA followed by visualization with alkaline phosphatase-conjugated secondary antibodies and development with ECL.

Currently, it is urgent but almost impossible to elucidate the function T3SS in *L. intracellularis* through genetic manipulation due to its obligate intracellular nature. In our previous study, we have constructed an in vitro secretion assay by using the heterologous *Yersinia* T3SS as an efficacious alternative to evaluate potential T3SS substrates [18]. In this study, we used the same method to determine whether LI1157–LI1159 are T3SS substrates. As described previously, LI1157–LI1159 were expressed in T3S-competent (WT) or -null (∆yscF) *Y. enterocolitica* as an HA-tagged protein under the control of an arabinose-inducible promoter, respectively. RplJ (an endogenous ribosomal protein) and YopE (an endogenous T3SS substrate) were used as the negative and positive controls, respectively. Cultured cells expressing the recombinant plasmid were centrifuged and fractionated into cell-free culture supernatants and *Yersinia*-containing whole-cell pellets. Immunoblot analysis of WT cultures demonstrated almost equal specific signals of LI1157–LI1159 in + Ca^2+^ (repressive) and − Ca^2+^ (inductive) cell pellet fractions (Figure 3). However, LI1158 and LI1159 were found in the supernatant under T3SS-inductive conditions but not under T3SS-repressive conditions, which is similar to the case of the positive control YopE. This result was not due to bacterial lysis, because the *Yersinia* cytoplasmic protein RplJ was detected in whole-cell pellet samples but not in the supernatant fractions (Figure 3). LI1158, LI1159, and the positive control YopE were not detected in the supernatant from the ∆yscF strain, which also validates that the secretion of LI1158 and LI1159 depends on a functional T3SS in *Yersinia* (Figure 3). Consistent with the known behavior of most T3S chaperones, LI1157 was detected in whole-cell pellet samples but not in the supernatant fractions.

To further identify the T3S signal of the LI1158 and LI1159 protein, we analyzed the secretion of the fusion protein consisting of the first 40 aa of LI1158 or LI1159 and YopE_ΔN15_ (LI1158_N40_-YopE_ΔN15_ or LI1159_N40_-YopE_ΔN15_). As a result, LI1158_N40_-YopE_ΔN15_ and LI1159_N40_-YopE_ΔN15_ were detected in the supernatant from WT cultures grown under T3SS-inductive but not under T3SS-repressive conditions (Figure 3). LI1158_N40_-YopE_Δ15_ and LI1159_N40_-YopE_ΔN15_ were not detected in the supernatant from the ∆yscF strain, which also confirms that the secretion of LI1158_N40_-YopE_Δ15_ and LI1159_N40_-YopE_ΔN15_ is dependent on a functional T3SS in *Yersinia* (Figure 3). Taken together, these results demonstrated that LI1158 and LI1159, but not LI1157, were exported by the *Yersinia* T3SS, with the secretion signal being located at the N terminus (aa 1–40).

### LI1158 is non-toxic to yeast cells and exists as a stable oligomer/monomer in yeast and mammalian cells

**Figure 4:**
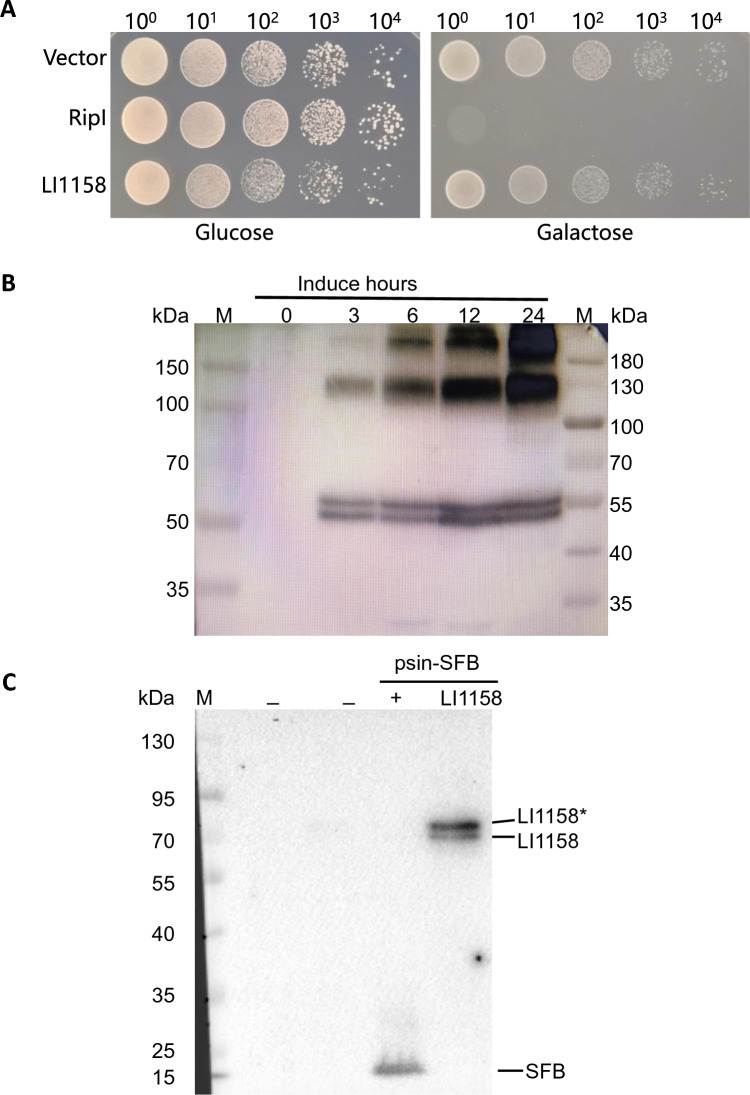
**LI1158 exists as oligomers/monomers in yeast and mammalian cells, respectively. A** Expression of recombinant LI1158 is non-toxic in yeast. W303-1A yeast strains carrying the yeast expression vector pRS416-GAL1, expressing either empty vector or LI1158 with an N-terminal 3 × Flag, were grown overnight in repressing medium (2% glucose). Cultures were then normalized to OD_600_ 1.0, and serial tenfold dilutions were grown at 30°C for 2 and 3 days in repressing and inducing medium (2% galactose and 1% raffinose), respectively. W303-1A yeast carrying the yeast expression vector pYES2/NT-RipI was used as the positive control. **B** LI1158 exists as oligomers in yeast cells. Induction of 3 × Flag-LI1158 expressions at different time points were verified by Western blotting using an anti-Flag antibody. **C** LI1158 exists as monomers in mammalian cells. Immunoblots of extracts of HEK293T cells transfected with psin-EF1-puro-SFB or pSIN-EF1-puro-SFB-LI1158 for 48 h. Cell lysates were probed with the anti-Flag tag antibody. Asterisks denote some unknown modification bands.

The bioinformatic data and *Yersinia* secretion assay suggested that LI1158 might be the major hydrophobic translocon protein of *L. intracellularis* T3SS. Expression of T3SS major translocon proteins such as CPn0809 from *Chlamydia pneumoniae* was reported to be harmful to yeast cells [22]. To determine whether LI1158 also inhibits yeast growth, flag-tagged LI1158 was induced to be expressed in *S. cerevisiae* strain W303-1A. Unexpectedly, LI1158 did not cause growth inhibition, which was different from the positive control RipI, a virulent phytopathogenic effector protein [24] (Figure 4A).

To determine the expression level of LI1158, yeast cell lysates were prepared and immunoblotted with an anti-FLAG antibody. The results showed that when galactose was not added (t = 0), no band was detected and the cross reaction with the anti-flag antibody could be excluded. With the extension of expression, an individual monomer and DTT- and SDS-resistant oligomers (dimer or tetramer, the molecular weight of the 3 × flag tag fusion LI1158 protein monomer is 47 kDa) appeared (Figure 4B). Surprisingly, two bands of 53–55 kDa occurred in SDS-PAGE, indicating that LI1158 underwent modification when artificially expressed in yeast cells.

However, different from the expression pattern in yeast, when we transfected HEK293T cells with the psin-LI1158-SFB plasmid and subjected the eluted protein to SDS-PAGE/Western blot, LI1158 existed as a monomer (the molecular weight of the S-protein-2 × flag-SBP tag fusion LI1158 protein monomer is 56 kDa) (Figure 4C). Two bands of 70–75 kDa occurred in SDS-PAGE of HEK293T cell lysates, indicating that the monomer of LI1158 also underwent modification when artificially expressed in both mammalian cells.

### LI1158 and LI1159 self-interact and LI1159 interacts with LI1157

Previous research has indicated that translocon proteins interact with each other and each pair of major and minor translocon proteins share a common chaperone [7, 12]. Our bioinformatic analysis results also indicated that both LI1158 and LI1159 harbor the CC domains, which could be involved in the structural mechanisms of oligomerization. To further determine whether LI1158 and LI1159 have similar functional features to T3SS translocon proteins from other species, such as oligomerization, chaperone binding, and mutual interaction, we performed Y2H assays.

**Figure 5:**
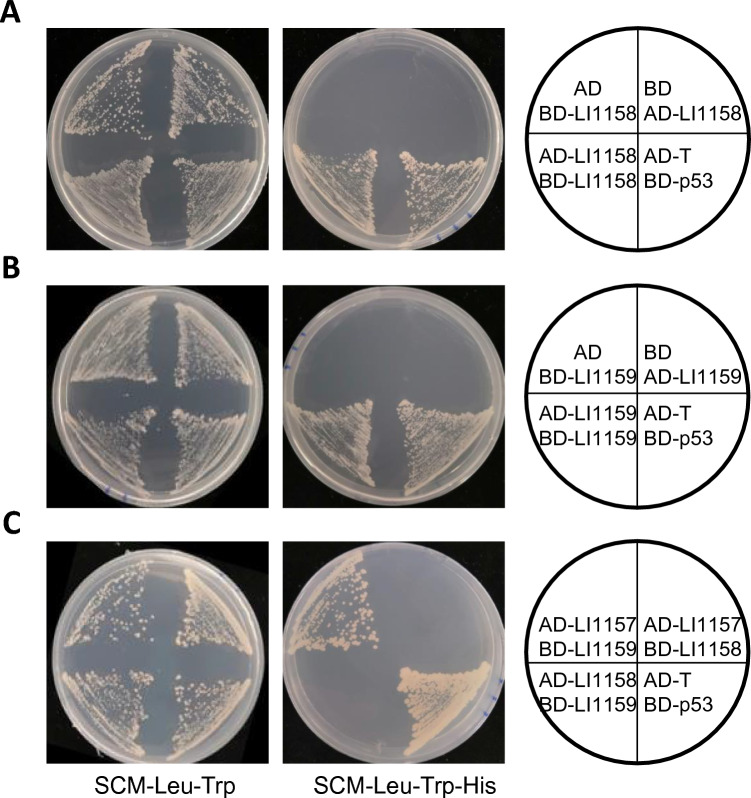
**LI1158 and LI1159 self-interact and LI1159 interacts with LI1157. A–C** Different combinations of plasmids (bait and prey) were transformed into yeast strain Y2H Gold, and then streaked on selective medium (Leu-, Trp-; growth control) and low stringency medium (Leu-, Trp-, His-), respectively. The plasmids pGBKT7-p53 (human protein p53) and pGADT7-T (large Tantigen of SV40) were co-transformed into yeast cells as positive control. Then, self-activation was carried out to overrule the interaction with vectors.

As shown in Figures 5A and B, Y2H gold yeast strains co-transformed with pGADT7-LI1158/pGBKT7-LI1158 or pGADT7-LI1159/pGBKT7-LI1159 plasmids grew on both plasmid selective medium and low-stringency selective medium, indicating that their interaction activates the expression of the Y2H reporter gene, though the activation was not as strong as that of the positive control (pGBKT7-p53/pGADT7-T). Surprisingly, the yeast cells co-transformed with pGADT7-LI1158/pGBKT7-LI1159 plasmids could not grow on the selective medium, suggesting that LI1158 and LI1159 have no interaction (Figure 5C). Yeast cells co-transformed with pGADT7-LI1157/pGBKT7-LI1159 could grow on the selective medium, but those co-transformed with pGADT7-LI1157/pGBKT7-LI1158 plasmid could not, indicating that LI1157 interacts with LI1159, but not with LI1158 (Figure 5C). Taken together, these results demonstrated that LI1158 or LI1159 is able to interact with itself and LI1159 can interact with the putative T3S-related class II chaperone LI1157.

### Expression of recombinant LI1159, but not LI1158, is toxic to *E. coli*

**Figure 6:**
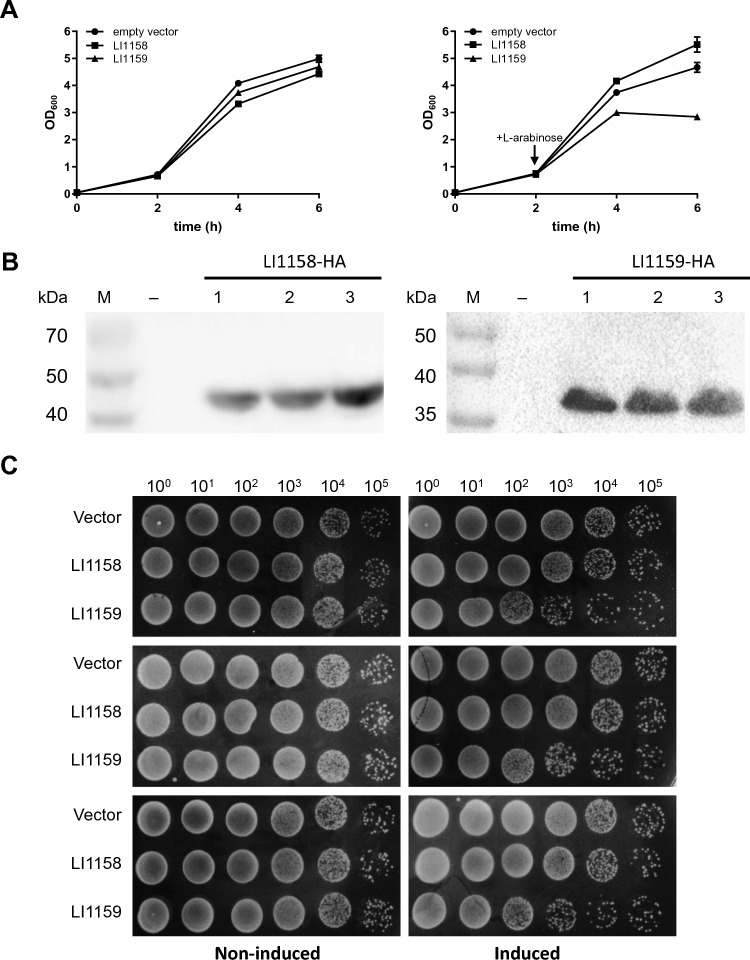
**Expression of LI1159 is toxic to *E. coli*. A** Growth curves of *E. coli* harboring different plasmid constructs under inducing or non-inducing conditions. Expression of LI1158-HA and LI1159-HA was induced by the addition of l-arabinose (+ l-arabinose, red mark). The mean of three independent replicates was used to create the growth curves by GraphPad Prism 7.0. **B** LI1158/LI1159-HA was expressed upon induction in *E. coli* cells. Induction of LI1158/LI1159-HA expressions were verified by Western blotting using an anti-HA antibody. **C** The toxic effect of LI1159 expression in *E. coli* was confirmed in serial dilution patch tests of *E. coli* transformants shown in **A**. Liquid cultures were grown for equal time periods and expression of proteins was induced by the addition of l-arabinose for 1 h. Ten microgram samples were taken and diluted ranging from 10^–1^ to 10^–4^. Dilutions were spotted onto solid LB-media with 1% glucose (repressing condition) and incubated overnight at 37°C.

Expression of T3SS major translocon proteins, such as CPn0809 from *C. pneumoniae* and YopB from *Yersinia*, was reported to be toxic to *E. coli* [15, 22]. To determine whether LI1158 and LI1159 also have a growth-inhibiting effect on *E. coli*, we utilized the LI1158-HA and LI1159-HA plasmid and tested them in comparison to the empty vector in a time-course experiment. Under non-inducing conditions, the growth rates of the three expression cultures were almost identical (Figure 6A). Upon induction of protein expression, the culture expressing either the empty vector or LI1158 protein continued to grow. In contrast, the growth of cultures expressing LI1159 protein was slow (4 h) or even stopped, indicating that LI1159 expression completely inhibited the growth of *E. coli* cells (Figure 6A). The LI1158-HA and LI1159-HA expression could be confirmed by using Western blot experiments (Figure 6B). To clarify whether LI1159 expression causes the growth arrest or actually kills the cells, we spotted cells from cultures after 1 h of protein expression and non-induced control cultures at different dilutions on plates (non-inducing medium). Cells from the three non-induced cultures showed an identical regular growth pattern on the medium. In contrast, *E. coli* cells induced to express LI1159 showed slower growth on the medium than cells expressing LI1158 and the empty vector (Figure 6C). Taken together, our findings demonstrated that LI1159 has a cytotoxic effect when expressed in *E. coli* cells, but LI1158 does not.

### Expression of recombinant LI1158 and LI1159 is non-toxic to *Y. enterocolitica* cells

**Figure 7:**
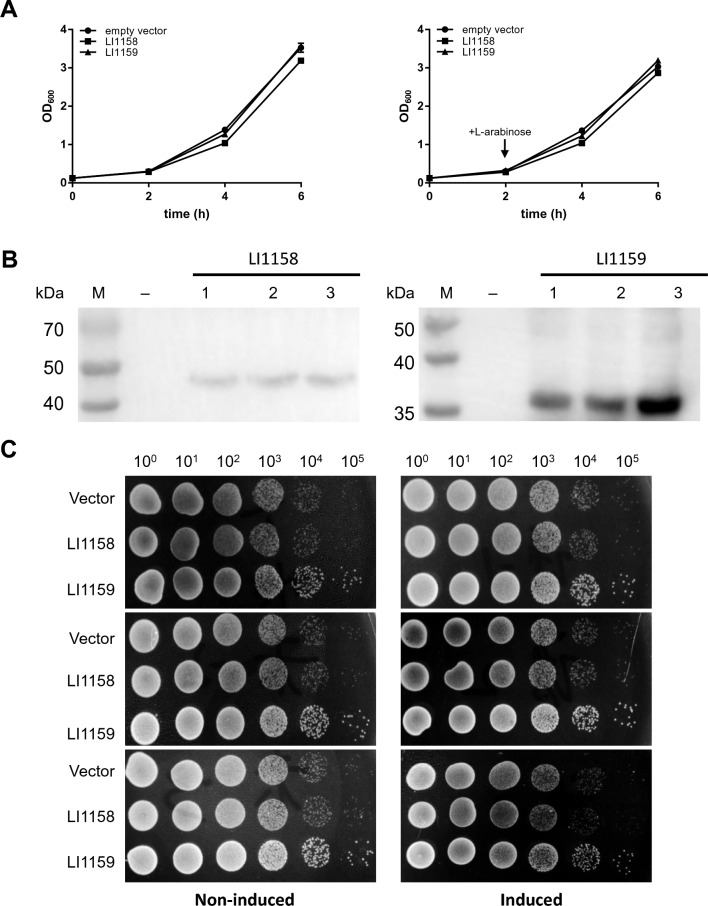
**Expression of recombinant LI1158/LI1159 is non-toxic for *Y. enterocolitica* cells.** The growth curves (**A**), Western blot experiment (**B**) and serial dilution patch test (**C**) in *Y. enterocolitica* cells were obtained in the same way as in *E. coli* cells.

Although LI1159, but not LI1158 was found to have a cytotoxic effect on *E. coli* cells, our *Yersinia* T3SS secretion assay indicated that LI1158/LI1159 is non-toxic to *Y. enterocolitica* cells (Figure 3), which prompted us to perform the time-course experiment and the serial dilution patch test in *Y. enterocolitica*. As expected, under both non-induced and induced conditions, the growth rates of the three expression cultures were almost identical (Figure 7A). Our western blot experiments also confirmed the expression of LI1158-HA and LI1159-HA in these growth curve experiments (Figure 7B). Cells from the three non-induced cultures and three induced cultures showed an identical regular growth pattern on plates (Figure 7C). Taken together, these results demonstrated that the expression of recombinant LI1158 and LI1159 is non-toxic to *Y. enterocolitica* cells.

## Discussion

Many Gram-negative bacteria pathogenic to plants and animals deploy T3SS to inject effector proteins into their hosts [7]. The finding that *L. intracellularis* may encode T3S components has stimulated particular interests in elucidating when and how *L. intracellularis* uses the T3S mechanism to deploy proteins to modulate host cell pathways like other Gram-negative pathogens [16]. However, it has been challenging to verify whether this secretion pathway is functional in *L. intracellularis* due to its obligate intracellular nature and genetic intractability. T3SS translocon is assembled by two hydrophobic membrane proteins, namely the major and minor translocon proteins at the tip of the T3SS needle, which form a pore in the plasma membrane of target cells. Currently, little information is available on the T3S components and translocon proteins of *L. intracellularis*.

A phylogenetic analysis of four conserved T3S apparatus proteins has revealed that the T3S apparatus of *Desulfovibrio vulgaris*, one of the three family members in Desulfovibrionaceae, belongs to the Ysc T3S apparatus of *Yersinia* spp., which is regarded as the archetype of T3SS on the plasmid [7]. *L. intracellularis,* another family member in Desulfovibrionaceae, possesses T3S components scattered throughout its genome (Table 3). Our BLASTP analysis revealed the sequence similarity between the orthologous proteins in the T3SS of *Y. enterocolitica* and *L. intracellularis*, further confirming that T3SS plays an important role in the intracellular pathogenesis of *L. intracellularis*.

In this study, we characterized the putative translocon proteins LI1158 and LI1159 in *L. intracellularis*, which are highly expressed in the enterocyte cytoplasm during infection [17]. Our bioinformatics analysis suggested the presence of common structural features of major translocon proteins, such as hydrophobic domains surrounded by CC structures, the SseC-like family domain, and the PxLxxP motif, in *L. intracellularis* LI1158 (Figure 2). However, LI1159, a putative minor translocon protein, only harbors the CC domain at its C terminus and has no TM. Considering its functions in the formation of a pore in the plasma membrane of target cells, further research is needed to detect and verify whether LI1159 contains hydrophobic domains.

Since translocon proteins are the substrate of T3SS, we employed the *Yersinia* T3SS as a heterogenous system to determine whether *L. intracellularis* LI1157–LI1159 are the substrate of T3SS. We found that LI1158 and LI1159, but not LI1157, are two novel substrates that have the potential to be secreted into culture supernatants, whose specific secretion signals located at the N terminus are recognized by *Y. enterocolitica* (Figure 3). Further research can be conducted to determine whether LI1158 and LI1159 have the capability of insertion into the target mammalian cell membrane by using *Y. enterocolitica* as a heterologous system.

Expression of T3SS major translocon proteins, such as CPn0809 from *C. pneumoniae*, was reported to be toxic to yeast cells [22]. Surprisingly, LI1158 showed no inhibitory effect on the growth of yeast cells in this study. Major translocon proteins are capable of forming homo- and/or hetero-oligomers [6]. In our *Yersinia* secretion assays, LI1158 did not form oligomers in the supernatant and the pellets. However, when heterogeneously expressed in *S. cerevisiae*, LI1158 existed as oligomers. When heterogeneously expressed in mammalian cells, LI1158 existed as monomers and underwent an unknown modification as in budding yeast. Further investigation is required to uncover the mechanism for the modification of LI1158 in yeast and mammalian cells.

A Y2H assay was performed to further verify whether LI1158 and LI1159 share common functional features with T3SS translocon proteins from other pathogenic species. We found that LI1158 and LI1159 are able to self-interact and LI1159 can interact with LI1157. However, LI1158 showed no interaction with LI1159 and LI1157. Given the importance of such interactions and their universality in other pathogens, these interactions need to be detected and verified in future experiments**.**

Finally, we analyzed the growth-inhibiting effect of LI1158 and LI159 on *E. coli* and *Y. enterocolitica* cells. Surprisingly, it was the minor translocon protein (LI1159) instead of the major translocon protein (LI1158) that led to *E. coli* cell death [15, 21]. These results also implied certain differences of *L. intracellularis* from other bacteria. Moreover, LI1159 did not inhibit the cell growth when expressed in *Yersinia*, which is totally different from its effect on *E. coli* cells.

Overall, despite certain differences from other bacteria, our results support the assumption that LI1158 and LI1159 are respectively the major and minor translocon proteins of *L. intracellularis* T3SS. Our findings improve understanding of the T3S components and will facilitate the development of more specific serological diagnostic assays and effective vaccines to control PE outbreak.

## Data Availability

The data supporting the findings of this study are available within the article, further inquiries can be directed to the corresponding author.

## References

[1] Lawson GH, McOrist S, Jasni S, Mackie RA (1993). Intracellular bacteria of porcine proliferative enteropathy: cultivation and maintenance in vitro. J Clin Microbiol.

[2] Karuppannan AK, Opriessnig T (2018). *Lawsonia intracellularis*: revisiting the disease ecology and control of this fastidious pathogen in pigs. Front Vet Sci.

[3] Vannucci FA, Gebhart CJ (2014). Recent advances in understanding the pathogenesis of *Lawsonia intracellularis* infections. Vet Pathol.

[4] Resende TP, Pereira CER, Daniel AGdS, Vasquez E, Saqui-Salces M, Vannucci FA, Gebhart CJ (2019). Effects of *Lawsonia intracellularis* infection in the proliferation of different mammalian cell lines. Vet Microbiol.

[5] Resende TP, Medida RL, Vannucci FA, Saqui-Salces M, Gebhart C (2020). Evaluation of swine enteroids as in vitro models for *Lawsonia*
*intracellularis* infection. J Anim Sci.

[6] Wagner S, Grin I, Malmsheimer S, Singh N, Torres-Vargas CE, Westerhausen S (2018). Bacterial type III secretion systems: a complex device for the delivery of bacterial effector proteins into eukaryotic host cells. FEMS Microbiol Lett.

[7] Cornelis GR (2006). The type III secretion injectisome. Nat Rev Microbiol.

[8] Wolf-Watz H, Portnoy DA, Bolin I, Falkow S (1985). Transfer of the virulence plasmid of *Yersinia pestis* to *Yersinia pseudotuberculosis*. Infect Immu.

[9] Juris SJ, Shao F, Dixon JE (2002). Yersinia effectors target mammalian signalling pathways. Cell Microbiol.

[10] Forsberg Å, Viitanen A-M, Skurnik M, Wolf-Watz H (1991). The surface-located YopN protein is involved in calcium signal transduction in *Yersinia pseudotuberculosis*. Mol Microbiol.

[11] da Cunha M, Milho C, Almeida F, Pais SV, Borges V, Maurício R, Borrego MJ, Gomes JP, Mota LJ (2014). Identification of type III secretion substrates of *Chlamydia trachomatis* using *Yersinia enterocolitica* as a heterologous system. BMC Microbiol.

[12] Dey S, Chakravarty A, Guha Biswas P, De Guzman RN (2019). The type III secretion system needle, tip, and translocon. Protein Sci.

[13] Davis AJ, Mecsas J (2007). Mutations in the *Yersinia pseudotuberculosis* type III secretion system needle protein, YscF, that specifically abrogate effector translocation into host cells. J Bacteriol.

[14] Sarker MR, Neyt C, Stainier I, Cornelis GR (1998). The Yersinia Yop virulon: LcrV is required for extrusion of the translocators YopB and YopD. J Bacteriol.

[15] Neyt C, Cornelis GR (1999). Role of SycD, the chaperone of the *Yersinia* Yop translocators YopB and YopD. Mol Microbiol.

[16] Pilar Alberdi M, Watson E, McAllister GEM, Harris JD, Paxton EA, Thomson JR, Smith DGE (2009). Expression by *Lawsonia intracellularis* of type III secretion system components during infection. Vet Microbiol.

[17] Vannucci FA, Foster DN, Gebhart CJ (2013). Laser microdissection coupled with RNA-seq analysis of porcine enterocytes infected with an obligate intracellular pathogen (*Lawsonia intracellularis*). BMC Genomics.

[18] Chen C, Dai Y, Yang Y, Zhu Z, Zhang Q, An X, Lai F (2022). *Lawsonia intracellularis* LI0666 is a new EPIYA effector exported by the *Yersinia enterocolitica* type III secretion system. Vet Res.

[19] Sorg I, Wagner S, Amstutz M, Müller SA, Broz P, Lussi Y, Engel A, Cornelis GR (2007). YscU recognizes translocators as export substrates of the Yersinia injectisome. EMBO J.

[20] Yang L, Lai F, He L, Lu Y, Zhong Q, Lai C, Dai Y (2019). LI1035, a putative effector secreted by Lawsonia intracellularis, targets the MAPK pathway and regulates actin organizationin yeast and mammalian cells. Vet Microbiol.

[21] Romano FB, Tang Y, Rossi KC, Monopoli KR, Ross JL, Heuck AP (2016). Type 3 secretion translocators spontaneously assemble a hexadecameric transmembrane complex. J Biol Chem.

[22] Engel AC, Herbst F, Kerres A, Galle JN, Hegemann JH (2016). The Type III secretion system-related CPn0809 from *Chlamydia pneumoniae*. PLoS One.

[23] Bulir DC, Waltho DA, Stone CB, Mwawasi KA, Nelson JC, Mahony JB (2014). *Chlamydia pneumoniae* CopD translocator protein plays a critical role in type III secretion (T3S) and infection. PLoS ONE.

[24] Deng MY, Sun YH, Li P, Fu B, Shen D, Lu YJ (2016). The phytopathogenic virulent effector protein RipI induces apoptosis in budding yeast *Saccharomyces cerevisiae*. Toxicon.

